# Screening, Molecular Identification and Degradation Characteristics of a Diflufenican-Degrading Bacterial Strain

**DOI:** 10.3390/toxics14080655

**Published:** 2026-07-25

**Authors:** Guangling Li, Lanfen Xie, Linling Lv, Runqiang Liu, Yanbing Wu, Jiangtao Li, Renhai Wu

**Affiliations:** 1 School of Plant Protection and Environment, Henan Institute of Science and Technology, Xinxiang 453003, China; lgl3298@126.com (G.L.);; 2 Guangxi State Farms Group Co., Ltd., Nanning 530023, China; 3 Institute of Plant Protection, Henan Academy of Agricultural Sciences, Zhengzhou 453000, China

**Keywords:** persistent herbicide, diflufenican-degrading bacteria, *Enterobacter hormaechei*, degradation characteristics

## Abstract

This study aims to identify microbial strain resources capable of degrading diflufenican and elucidate their degradation characteristics, with the goal of mitigating the phytotoxicity hazards associated with the prolonged use of this persistent herbicide. A degradation strain was isolated, purified, and screened from wheat field soils that had been subjected to diflufenican treatment using an enrichment culture method. The taxonomic classification of the strain was determined through a comprehensive analysis of its morphology, physiology, biochemistry, as well as its 16S rRNA gene sequence. The results demonstrated that the screened bacterial strain 88-1 could utilize diflufenican as its metabolic carbon source and was identified as *Enterobacter hormaechei*, a facultative anaerobe. The degradation efficiency of strain 88-1 on diflufenican was closely associated with cultivation time, the initial concentration of the herbicide, temperature, pH, and inoculation amount of the strain. Additionally, the degradation rate exhibits a positive correlation with the biomass of the strain. Under optimal conditions (40 mg/L diflufenican, 30 °C, pH 8.0, 10% inoculum), the highest observed degradation efficiency and viable cell density over the 120 h incubation period were 55.11% and 8.05 × 10^6^ CFU/mL, respectively. Furthermore, rapid biotransformation commenced within 24 h, yielding a cascade of metabolites, with 2-(3-(trifluoromethyl)phenoxy)pyridine-3-carboxamide identified as the primary metabolite. These findings suggest that strain 88-1 holds promise for the bioremediation of soils contaminated with diflufenican.

## 1. Introduction

**Figure 1:**
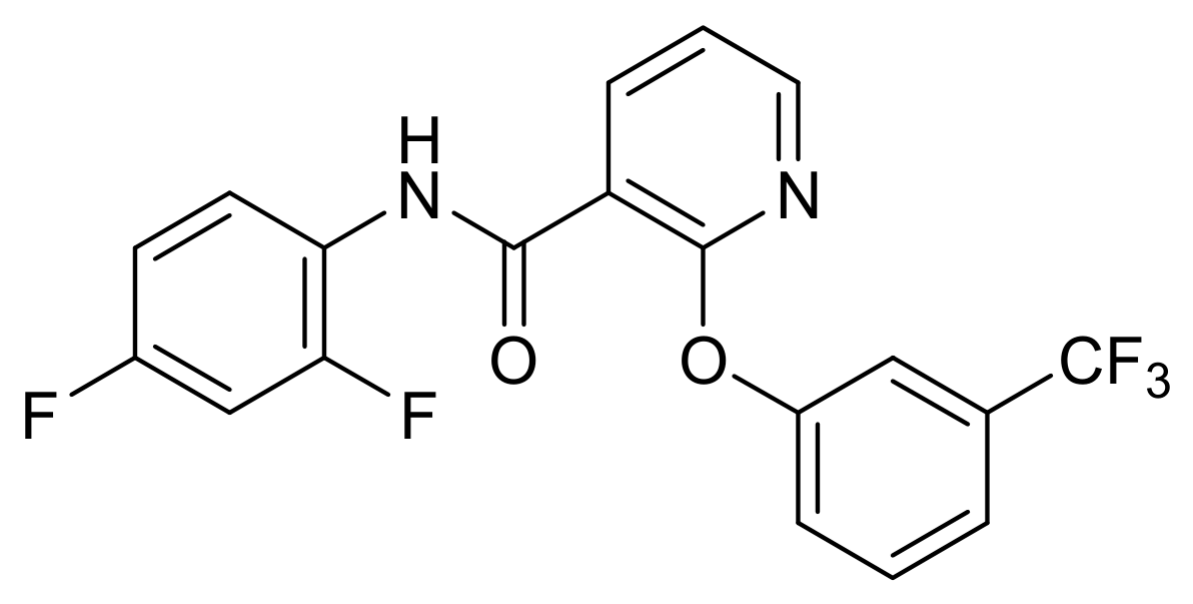
The structural formula of diflufenican.

Diflufenican is a pyridine-based amide herbicide developed by Rhone-Poulenc (France) in 1982, with the chemical name N-(2,4-difluorophenyl)-2-[3-(trifluoromethyl)phenoxy]pyridine-3-carboxamide (chemical structure presented in Figure 1). This compound exerts its herbicidal activity by inhibiting carotenoid biosynthesis in plants, thereby disrupting the protective function of carotenoids against photooxidative damage and leading to chlorophyll degradation and photobleaching of green tissues [1]. The differential selectivity between crops and weeds is primarily attributed to variations in uptake, translocation, and metabolic detoxification processes [2]. Diflufenican is extensively used for the control of annual grass weeds and certain broadleaf weed species in cereal crops such as maize, wheat, and barley. At present, Europe remains its largest established market, while the Asia-Pacific region represents the fastest-growing market segment, and North America exhibits emerging growth potential [3]. The compound exhibits high environmental stability: its half-life ranges from 60 to 180 days in natural aquatic systems and from 14 to 35 weeks in agricultural soils [4]. These persistence profiles indicate that its persistence in soil may be jointly influenced by factors such as soil organic matter and microbial activity. In recent years, it has emerged as an herbicide of significant interest, with 66 formulated products currently registered specifically for use in wheat cultivation [5]. Of particular concern, diflufenican exhibits high environmental persistence due to its resistance to hydrolysis, photodegradation [3], and microbial degradation in soil [6], resulting in prolonged soil residue that may induce phytotoxic effects in rotational crops, including oilseed rape, cucumber, rice, and tomato. Furthermore, residual diflufenican has been shown to significantly alter the composition and functional activity of soil microbial communities [7], and poses long-term ecological risks to aquatic systems [8,9]. Given these environmental implications, the issue of diflufenican residue accumulation in agricultural environments warrants urgent attention, and appropriate risk mitigation and remediation strategies should be implemented.

Microbial degradation constitutes a safe and effective strategy for the remediation of pesticide residues. In recent years, numerous studies have reported the isolation of bacterial strains capable of degrading acetamide herbicides—such as acetochlor, metolachlor, pretilachlor, and butachlor—and their application in the bioremediation of contaminated soils. For example, Dong et al. [10] demonstrated that *Serratia marcescens* AB1 exhibits high degradation efficiency toward acetochlor, reducing its half-life from 80.81 days to 13.67 days in sterilized soil and from 146.27 days to 12.77 days in non-sterilized soil, while simultaneously enhancing soil enzyme activities. Kaur et al. [11] isolated *Bacillus altitudinis* A16 from coal tar-contaminated soil, a strain capable of utilizing butachlor as its sole carbon source. Under minimum cell density conditions, it degraded 90% of 50 mg/L butachlor within 5 days, with a degradation rate constant of 0.02 h^−1^ and a half-life of 34.65 h, indicating considerable potential for soil remediation applications. Zhao [12] isolated three *Rhodococcus* strains—MET-6, CMEPA-1, and EA-4—from sludge collected at a metolachlor manufacturing facility. Among these, strain MET-6 achieved over 50% degradation of alachlor, acetochlor, metolachlor, pretilachlor, and butachlor within 4 days under pure culture conditions, demonstrating both high efficiency and broad-spectrum degradative capability. Zhang et al. [13] identified *Bacillus cereus* LGY06, which degraded 75.7% of 50 mg/L pretilachlor within 7 days in a pure culture system. In laboratory-scale simulated soil experiments, inoculation with LGY06 significantly accelerated the degradation of pretilachlor residues. Compared to non-inoculated controls, the half-life of pretilachlor was reduced by 79.5%, 36.6%, and 41.1% in sterilized soil, non-rhizosphere soil, and rhizosphere soil, respectively, suggesting that this strain holds promise for practical application in the remediation of herbicide-contaminated environments.

A systematic literature review revealed that, to date, only one peer-reviewed conference publication reporting bacterial strains capable of degrading diflufenican has been identified in the international scientific literature. In 2022, Ksiazek-Trela et al. [8] evaluated the efficacy of three commercially available complex microbial inoculants—comprising 23 bacterial strains including *Bifidobacterium*, *Streptococcus*, and *Bacillus*—in degrading diflufenican residues in soil. The results indicate that these formulations not only failed to enhance herbicide degradation but also significantly inhibited the degradation rate in soil. The authors attributed this inhibitory effect to the low pH of the experimental soil, which likely enhanced the chemical stability of diflufenican residues and consequently impeded their natural degradation. In 2024, Ksiazek-Trela et al. [14] reported the isolation of four bacterial strains—A1, A2, C1, and D1—from soil samples exhibiting the capacity to degrade diflufenican. Among these, strain D1 exhibited the highest degradation efficiency: under liquid culture conditions with an initial diflufenican concentration of 220 mg L^−1^, it achieved 70% degradation after 21 days of incubation—coinciding with the onset of the stationary phase. In sterilized soil, D1 degraded 17% of the herbicide residue over a 28-day experimental period. Furthermore, the study identified a synergistic degradation effect among the four strains; in liquid culture, the mixed consortium achieved a degradation rate of 75%, while in sterile soil, the degradation rate increased from 17% with individual strains to 29% in the combined treatment. Given the persistent phytotoxic risks associated with diflufenican—stemming from its prolonged residual activity in agricultural soils—there is an urgent need to identify efficient microbial agents for its biodegradation. To address this gap, soil samples were collected from wheat fields with a documented history of long-term diflufenican application. Using enrichment culture coupled with sequential acclimation, bacterial strains capable of degrading diflufenican were successfully isolated. Isolates were taxonomically characterized through morphological observation, physiological and biochemical testing, and 16S rRNA gene sequencing; their degradation performance was then systematically evaluated under controlled conditions.

## 2. Materials and Methods

### 2.1. Experimental Instruments

The primary instruments used in this study include an Agilent 1260 HPLC system equipped with a binary pump, vacuum degasser, column oven, autosampler, and variable-wavelength ultraviolet detector (Agilent Technologies, Santa Clara, CA, USA); an Agilent 7890B-5977B GC-MS system (Agilent Technologies, Santa Clara, CA, USA); an LDZX-50KBS vertical autoclave (Shen’an Medical Instrument Co., Ltd., Shanghai, China); an SW-CJ-2F vertical laminar flow hood (Puwote Purification Equipment Co., Ltd., Suzhou, China); and a THZ-82A constant temperature orbital shaker (Yiheng Scientific Instrument Co., Ltd., Shanghai, China). All instruments were calibrated according to manufacturer specifications and maintained on a regular schedule to ensure data accuracy and experimental reproducibility.

### 2.2. Chemicals and Reagents

The chemicals and reagents used were as follows: 98.0% diflufenican analytical standard (Alta Technology Co., Ltd., Tianjin, China); 95% diflufenican technical grade material (Guangda Biotechnology Co., Ltd., Jiangsu, China); and chromatographic-grade acetonitrile (Mreda Medical Technologies Inc., Beijing, China). All reagents were used as received without further purification.

### 2.3. Soil Sample Collection

Soil samples were collected from the arable layer (0–15 cm) of wheat fields with a documented history of repeated diflufenican applications over the preceding five years. Composite soil samples were collected in triplicate from randomly selected subplots within the field to ensure representativeness and analytical reproducibility. Prior to sampling, surface litter and plant residues were carefully removed. Samples were transferred to sterile polyethylene bags, transported to the laboratory on ice, and stored at 4 °C in the dark until further processing.

### 2.4. Culture Medium Formulation

Mineral Salt Medium (MSM): MSM was prepared by dissolving 1.5 g NH_4_NO_3_, 0.5 g KH_2_PO_4_, 1.5 g K_2_HPO_4_, 0.5 g NaCl, and 0.2 g MgSO_4_·7H_2_O in 1000 mL of deionized water. The solution was stirred until fully dissolved, adjusted to pH 7.2–7.4, and autoclaved at 121 °C for 20 min. This medium was used for the enrichment and isolation of diflufenican-degrading microorganisms.

Luria–Bertani Medium (LBM): LBM was prepared by dissolving 10 g tryptone, 5 g yeast extract, and 5 g NaCl in 1000 mL of deionized water, and autoclaving at 121 °C for 20 min.

For solid medium preparation, 20 g agar was added per liter after sterilization and while cooling to approximately 50 °C, followed by thorough mixing and pouring into sterile Petri dishes.

### 2.5. Experimental Methods

#### 2.5.1. Sample Preparation and Quantitative Determination

The bacterial suspension of herbicide-degrading strains was vortexed for 3 min to ensure complete homogenization. An aliquot of 1 mL was accurately transferred into a 5 mL polytetrafluoroethylene centrifuge tube, followed by the addition of 1 g sodium chloride and 2 mL acetonitrile. The mixture was subjected to vigorous vortex extraction for 3 min and then allowed to stand for 5 min to facilitate phase separation. Subsequently, 700 μL of the upper organic layer was collected and filtered through a 0.22 μm syringe filter before transfer to an autosampler vial for quantitative analysis by HPLC-VWD.

Chromatographic separation was performed on an Ultimate XB-C18 column (4.6 mm × 250 mm, 5 μm) maintained at 35 °C. The mobile phase consisted of methanol–water (85:15, *v*/*v*) delivered at a constant flow rate of 1.0 mL/min. A 5 μL aliquot was injected, and detection was conducted at 285 nm.

#### 2.5.2. Enrichment, Isolation, and Purification of Strains

Seventy-five soil samples (5 g each), collected from wheat fields with a history of diflufenican application, were individually transferred to 250 mL conical flasks containing 45 mL of inorganic salt medium supplemented with 5 mg/L diflufenican.

Enrichment cultures were incubated at 30 °C under shaking at 160 r/min for 5 days to establish preliminary microbial adaptation. Subsequently, 10% (*v*/*v*) aliquots of each culture were transferred into fresh inorganic salt medium amended with stepwise-increasing diflufenican concentrations (10, 20, 40, and 80 mg/L), with serial subculturing performed every 5 days to drive directional enrichment toward high-degradation phenotypes. Upon completion of the enrichment regime, enriched cultures were streaked onto LB agar plates and incubated at 30 °C for 48 h to isolate and purify single colonies. Individual colonies were picked, subjected to subsequent expansion culture, and evaluated for diflufenican degradation efficiency using the HPLC-VWD method detailed in Section 2.5.1 to obtain efficient degrading strains.

#### 2.5.3. Strain Identification

Morphological observations and physiological and biochemical characterization, as specified in Table 1, were performed on the efficiently degrading bacterial strains selected according to authoritative testing methods [15]. Total genomic DNA was extracted from the bacterial cultures using the Ezup Column-based Bacterial Genomic DNA Extraction Kit. The 16S rRNA gene fragment was amplified via PCR using the universal primers 27F (5′-AGA GTT TGA TCM TGG CTC AG-3′) and 1492R (5′-GGT TAC CTT GTT ACG ACT T-3′). The resulting PCR products were purified and sequenced. The obtained sequences were submitted to the NCBI GenBank database for BLAST 2.15.0 analysis to assess sequence homology. Based on the alignment results, a phylogenetic tree was constructed using MEGA 12.1 based on sequence alignment results. In brief, multiple sequence alignment was conducted using MUSCLE and ModelFinder was applied to screen for the optimal substitution model (GTR + I + G) based on the Akaike Information Criterion (AIC). Bootstrap analysis with 1000 replicates was performed to evaluate branch reliability. The sequence of *Budvicia aquatica* strain Eb 13 (NR 025332) was selected as the outgroup to root the phylogenetic tree.

#### 2.5.4. Degradation Characteristics

##### Determination of Degradation and Growth Kinetics of Candidate Strains

Candidate strains were inoculated into LB liquid medium and pre-cultured at 30 °C and 160 r/min for 4 h to achieve active growth. Subsequently, the seed culture was transferred into 50 mL of inorganic salt medium containing 40 mg/L diflufenican at a 10% (*v*/*v*) inoculum size and incubated under the same conditions with continuous shaking for 168 h. Samples were collected at 24 h intervals. Bacterial cell concentrations were determined using the spread plate method, while the residual concentration of diflufenican in the culture medium was quantified by HPLC-VWD. This study systematically incorporated controls for abiotic degradation pathways—including photolysis, hydrolysis, and cellular adsorption—during experimental design and execution. All degradation rate data were corrected against uninoculated diflufenican-containing medium controls to ensure accurate attribution of degradation activity to strain 88-1. The degradation rate was calculated according to Equation (1). All experiments were performed in quadruplicate to ensure statistical reliability.

(1) $$Degradation\;rate\;(\%)\;=\frac{Concentration\;of\;the\;control\;group-Concentration\;in\;the\;treatment\;group}{Concentration\;of\;the\;control\;group}\times 100$$

##### Optimization of Degradation Conditions for Candidate Strains

To determine the optimal culture temperature, medium pH, inoculum size, and substrate concentration for subsequent experiments, a sequential optimization approach was employed. First, under fixed conditions of 30 °C, pH 8.0, and 10% (*v*/*v*) inoculum size, substrate concentrations of 10, 20, 40, 80, and 160 mg/L were tested to evaluate their effects on degradation efficiency and identify the optimal substrate concentration. Subsequently, using the optimal substrate concentration, pH 8.0, and 10% inoculum size, cultures were incubated at 20, 25, 30, 35, and 40 °C to determine the most favorable temperature for degradation. Next, with the previously optimized substrate concentration and temperature, the inoculum size was maintained at 10%, while the medium pH was adjusted to 5.0, 6.0, 7.0, 8.0, and 9.0 to ascertain the optimal pH. Finally, under the above-established optimal conditions, inoculation volumes of 1%, 3%, 5%, 10%, and 15% (*v*/*v*) were evaluated to determine the optimal inoculum size. All treatments were conducted in flasks agitated at 160 r/min for 120 h, with non-inoculated culture medium serving as the blank control. Each experiment was performed in quadruplicate. The residual concentration of diflufenican was quantified using the HPLC-VWD method described in Section 2.5.1, and the degradation rate was calculated according to Equation (1). Bacterial growth was simultaneously monitored.

##### Determination of the Metabolic Capability of Candidate Strains

Under the optimized degradation conditions established in Section 2.5.2, candidate strains were co-cultured with diflufenican for 24 h. Subsequently, 2 mL of well-homogenized bacterial suspension was transferred into a 50 mL polytetrafluoroethylene centrifuge tube, followed by the addition of 1 g sodium chloride and 10 mL acetonitrile. The mixture was vortexed vigorously for 3 min to facilitate extraction and then allowed to stand for salt-induced phase separation. Upon complete phase stratification, 1 mL of the upper organic layer was passed through a 0.22 μm syringe filter and transferred to an autosampler vial for GC-MS qualitative analysis. An Agilent DB-35 MS capillary column (30 m × 250 μm, 0.25 μm film thickness) was employed for chromatographic separation. The GC inlet temperature was maintained at 260 °C, and high-purity helium was used as the carrier gas at a constant flow rate of 1.0 mL/min. The oven temperature program was set as follows: held at 50 °C for 1 min, increased to 180 °C at 150 °C/min, held for 1 min, then further ramped to 260 °C at 10 °C/min, and finally held for 10 min. The transfer line was set to 240 °C, the EI source operated at 230 °C, and the quadrupole detector at 150 °C. Mass spectra were acquired in full-scan mode over the range of m/z 40–450. A 1 μL sample was injected in split mode with a split ratio of 10:1.

### 2.6. Data Processing

All data were initially organized using Microsoft Excel 2020 and subsequently analyzed by one-way analysis of variance (One-Way ANOVA) using SPSS 26.0 software. Significant differences among means were determined using Duncan’s multiple range test at a significance level of α = 0.05.

## 3. Results

### 3.1. Performance Parameters of Chromatographic Analysis Methods

#### 3.1.1. Linearity

Under the chromatographic conditions specified in Section 2.5.1, a calibration series of diflufenican standard solutions (0.1–50 mg/L) was analyzed, generating a linear quantitative calibration curve described by the equation y = 8.4352x + 0.2295 (R^2^ = 0.9999).

#### 3.1.2. Accuracy and Sensitivity

According to the sample preparation protocol described in Section 2.5.1, recovery experiments were performed on bacterial suspension samples fortified with diflufenican at three concentration levels (1.0, 0.5, and 0.1 mg/kg). The mean recoveries ranged from 87.3% to 92.6%, with relative standard deviations ≤ 6.5%. The method detection limit (LOD) and method quantification limit (LOQ), determined based on signal-to-noise ratios of 3 and 5, respectively, were 0.0072 mg/L and 0.024 mg/L.

### 3.2. Identification of Candidate Strains

A total of 350 bacterial strains were isolated and purified from the enrichment culture. Based on their diflufenican degradation capacity, strain 88-1 was selected for further characterization due to its superior degradation performance. The strain was systematically identified using morphological, physiological and biochemical tests, as well as molecular biological methods.

#### 3.2.1. Morphological Identification

**Figure 2:**
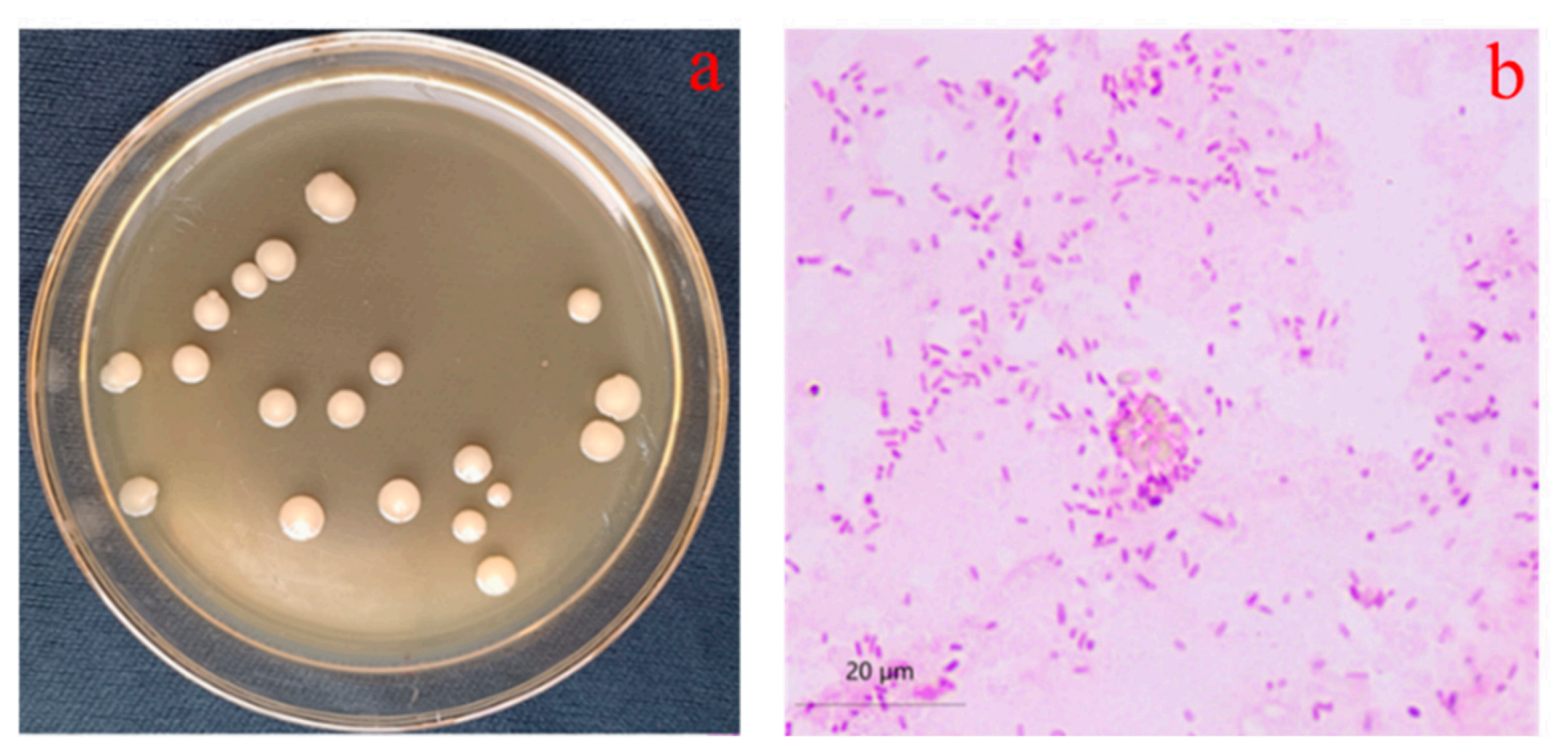
Bacterial colony morphology of strain 88-1 ((**a**). Morphology of single colonies of strain 88-1 on LB agar plates; (**b**). Gram staining of strain 88-1).

Strain 88-1 was inoculated onto LB solid medium via spot inoculation and incubated at 30 °C for 36 h. Colony morphology was examined (Figure 2), revealing milky-white, raised colonies with a moist surface, tightly adherent to the agar, neat margins, and dense consistency, with diameters ranging from 4.5 mm to 6.8 mm. Gram staining revealed red-stained cells, indicating a rod-shaped, Gram-negative bacterium.

#### 3.2.2. Physiological and Biochemical Characterization

The physiological and biochemical characteristics of strain 88-1 were presented in Table 1. The results show that strain 88-1 is unable to hydrolyze starch but can slowly liquefy gelatin; the methyl red (MR) test is negative, whereas the Voges-Proskauer (VP) test is positive; it does not produce H_2_S from thiosulfate; it can utilize citrate as the sole carbon and energy source; and it does not produce deoxyribonuclease.

#### 3.2.3. Molecular Biological Characteristics

**Figure 3:**
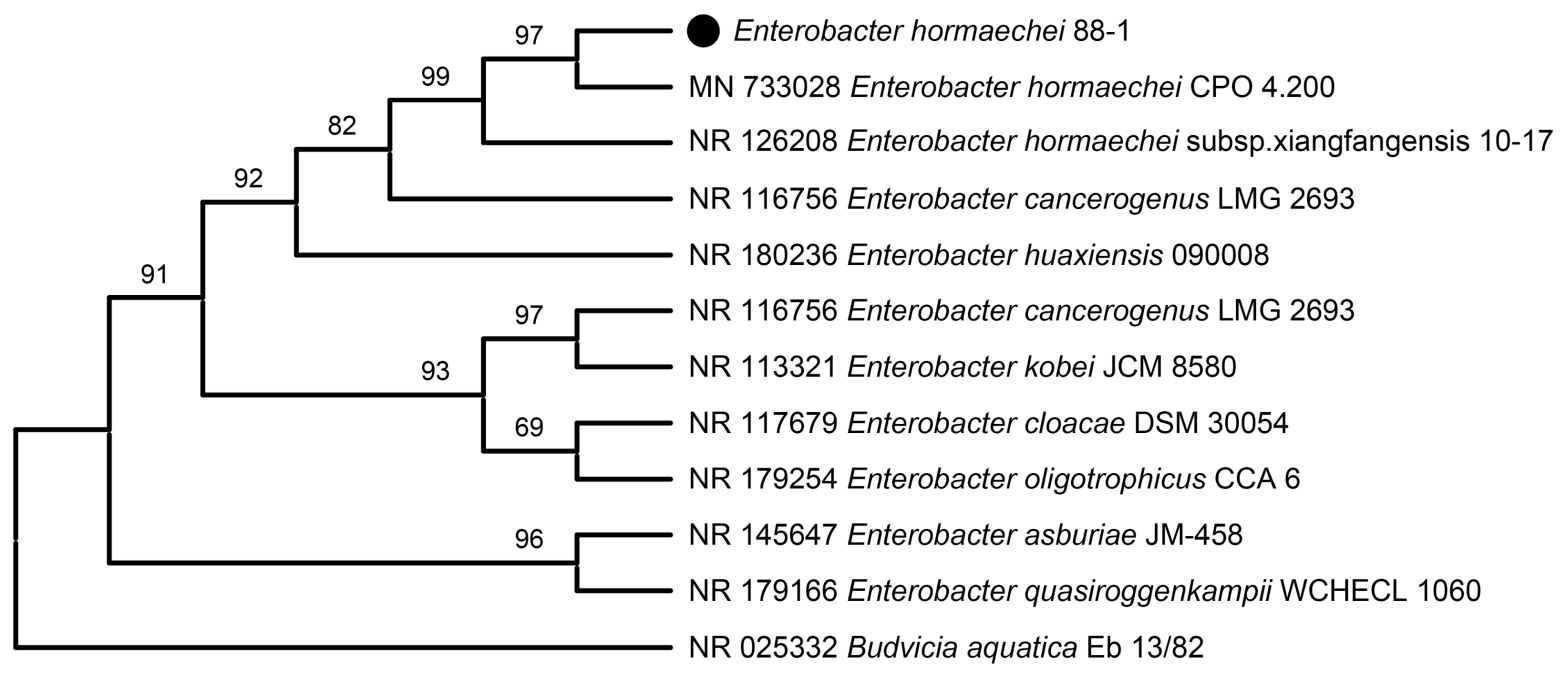
Phylogenetic tree based on 16S rRNA of strain 88-1 and its related species.

The genomic DNA of strain 88-1 was extracted, and the 16S rRNA gene fragment was amplified by PCR. The resulting 1478 bp sequence was analyzed using the Blastn program against the GenBank database (see Table 2), revealing the highest sequence similarity to Enterobacter hormaechei. Based on the bacterial morphological characteristics shown in Figure 2 and the physiological and biochemical traits listed in Table 1, strain 88-1 is classified within the genus *Enterobacter*, as a facultative anaerobe, and identified as *Enterobacter hormaechei*. The phylogenetic tree reconstruction results are presented in Figure 3.

### 3.3. Degradation Characteristics of Strain

#### 3.3.1. Relationship Between Growth and Degradation Capacity of Strain 88-1

**Figure 4:**
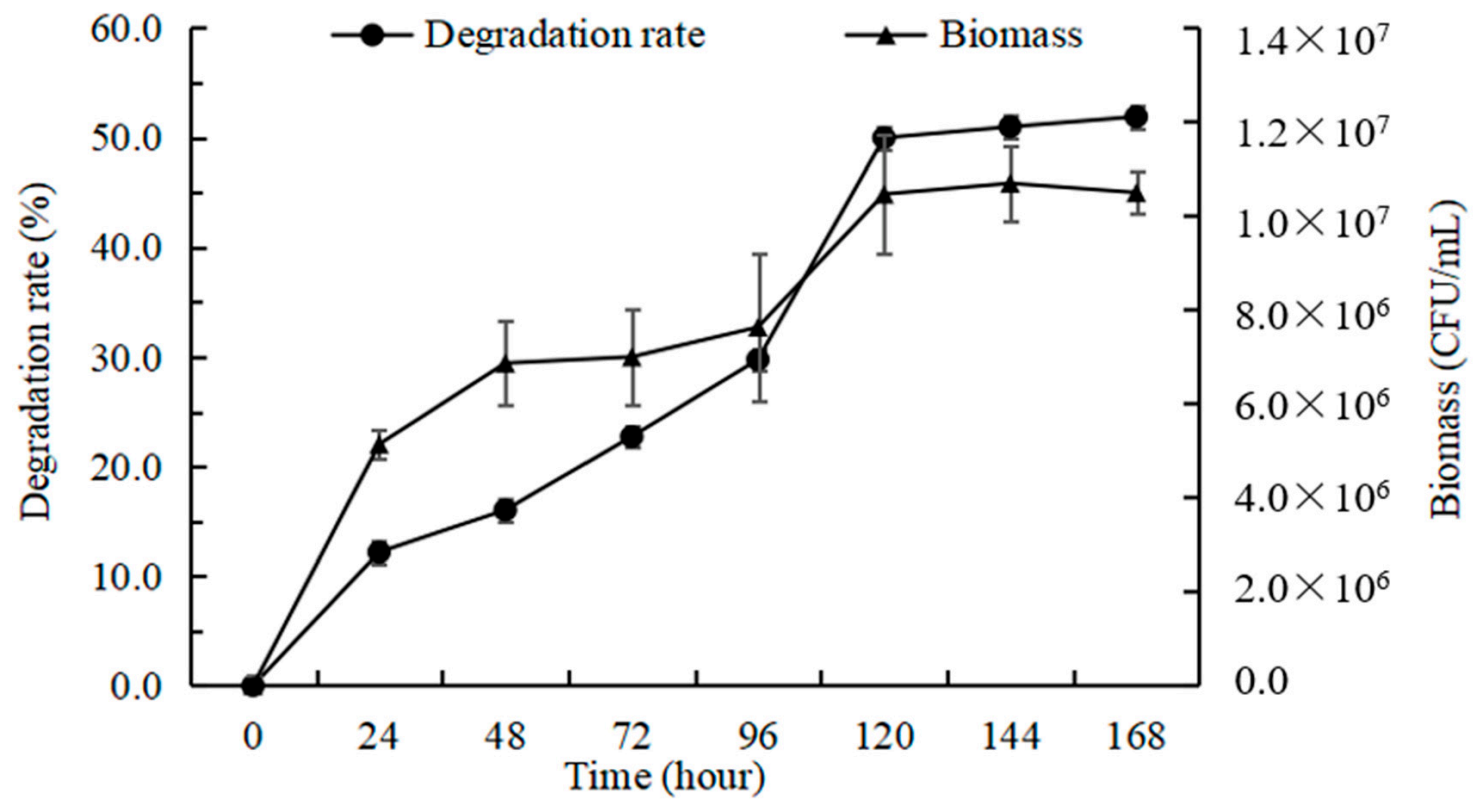
The dynamic in the degradation rate of diflufenican and the growth of strain 88-1 over the course of cultivation time.

Strain 88-1 utilizes diflufenican as a metabolic carbon source for growth and reproduction, and its growth rate is positively correlated with its degradation capacity within a defined incubation period. As illustrated in Figure 4, the degradation rate of diflufenican increases gradually with increasing biomass of strain 88-1. In inorganic salt medium supplemented with an initial concentration of 40 mg/L diflufenican, cell density increased from 5.14 × 10^6^ CFU/mL to 7.63 × 10^6^ CFU/mL between 24 h and 96 h of cultivation, accompanied by a corresponding rise in degradation efficiency from 12.17% to 29.76%. At 120 h, the bacterial density quantified via plate colony-forming unit (CFU) enumeration reached 8.05 × 10^6^ CFU/mL, concomitant with a diflufenican degradation rate of 55.11%. Between 120 h and 168 h, neither bacterial density nor degradation rate exhibited statistically significant change, confirming that strain 88-1 had attained a degradation plateau for diflufenican.

#### 3.3.2. Effects of Initial Diflufenican Concentration and Bacterial Growth on the Degradation Capacity of Strain 88-1

**Figure 5:**
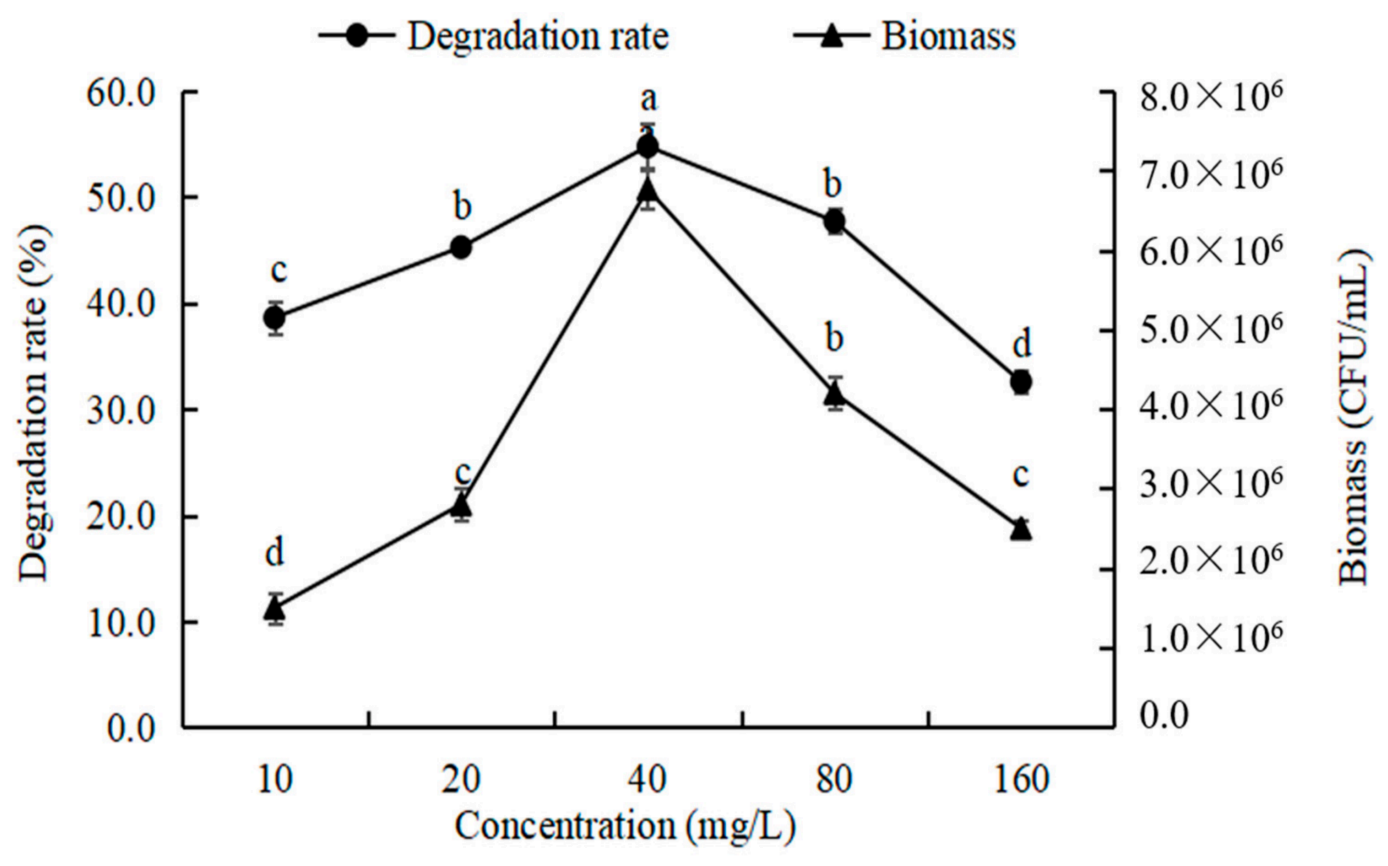
Effects of initial diflufenican concentration on the degradation rate and growth of strain 88-1 (Different lowercase letters indicated significant differences (α = 0.05) analyzed by Duncan’s multiple range test).

Diflufenican serves as a metabolic carbon source for strain 88-1, and its concentration in the culture medium reflects the availability of carbon supply. Experimental results show that under conditions of 30 °C, pH 8.0, and 10% inoculum size, when the initial diflufenican concentration ranges from 10 mg/L to 40 mg/L, the cell density of strain 88-1 increases significantly from 1.50 × 10^6^ CFU/mL to 6.77 × 10^6^ CFU/mL with increasing concentration, accompanied by a significant rise in degradation efficiency from 38.62% to 54.78%. However, when the initial concentration exceeds 40 mg/L and reaches up to 160 mg/L, both bacterial growth and degradation rate decline progressively with further concentration elevation. At a concentration of 160 mg/L, both cell density and degradation rate are markedly lower than their respective peak values (see Figure 5). This trend is likely attributable to the toxic effect of high diflufenican concentrations on strain 88-1, leading to inhibition of its metabolic activity and growth capacity.

#### 3.3.3. Effects of Cultivation Temperature and Strain 88-1 Growth on the Degradation Capacity

**Figure 6:**
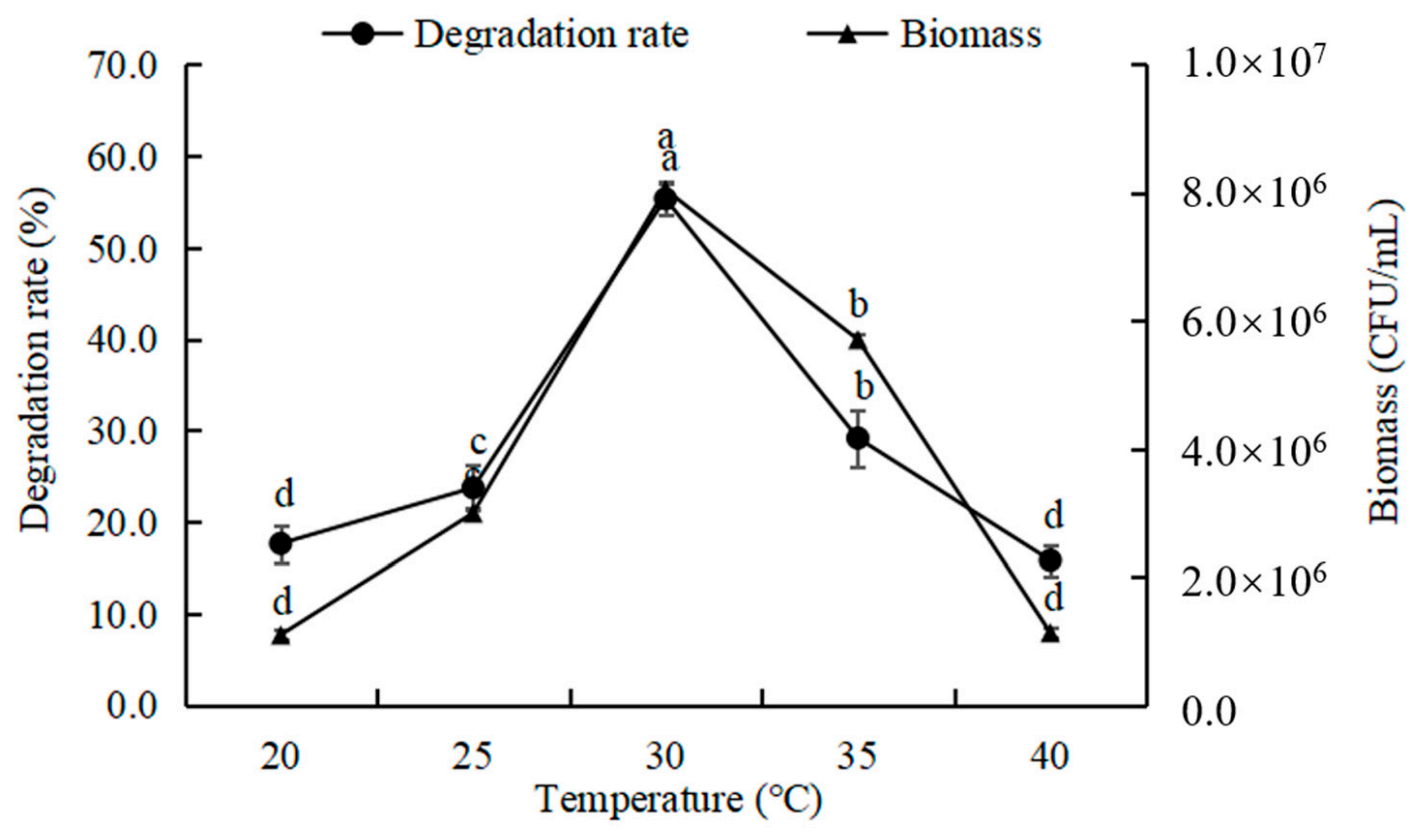
Effects of temperature on the degradation rate of diflufenican and growth of strain 88-1 (Different lowercase letters indicated significant differences (α = 0.05) analyzed by Duncan’s multiple range test).

Under conditions of an initial diflufenican concentration of 40 mg/L, a medium pH of 8.0, and an inoculum size of 10%, experimental results across different cultivation temperatures indicate that at 20 °C and 40 °C, both the cell density of strain 88-1 and its degradation capacity for diflufenican are relatively low, suggesting that suboptimal or excessive temperatures are unfavorable for biomass accumulation and inhibit the strain’s degradation ability. At 30 °C, after 168 h of cultivation, both cell density and degradation rate reach their maximum values, reaching 8.05 × 10^6^ CFU/mL and 55.36%, respectively. In contrast, at 35 °C, both strain 88-1 growth and degradation efficiency decline significantly. Overall, within the temperature range of 20 °C to 40 °C, the growth of strain 88-1 and its strain 88-1 degradation rate exhibit a unimodal trend, initially increasing with temperature before subsequently decreasing (see Figure 6).

#### 3.3.4. Effects of Medium pH and Growth of Strain 88-1 on Its Degradation Capacity

**Figure 7:**
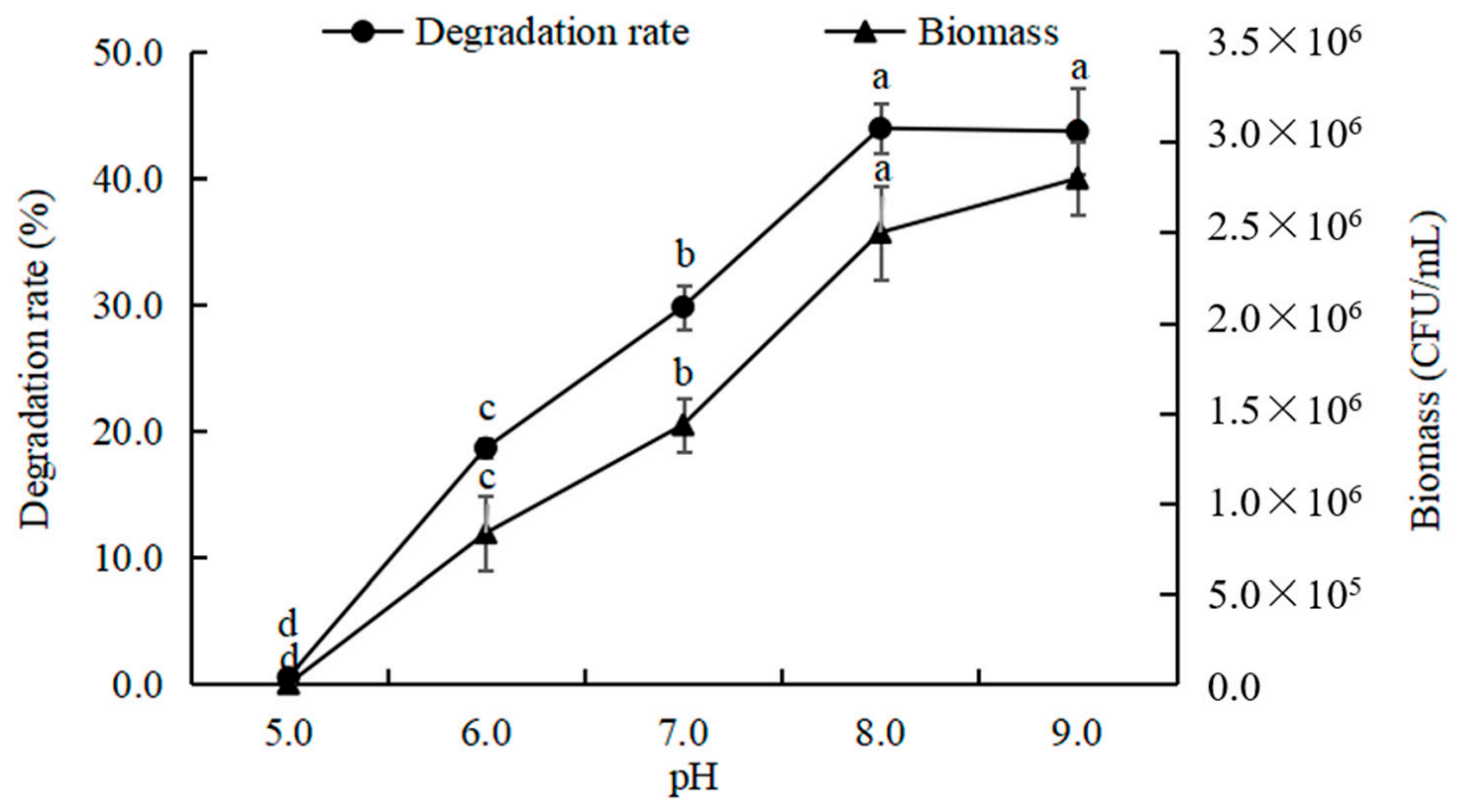
Effects of pH on the degradation rate of diflufenican and growth of strain 88-1 (Different lowercase letters indicated significant differences (α = 0.05) analyzed by Duncan’s multiple range test).

Medium pH is a critical factor affecting microbial metabolism and growth, and different bacterial strains generally exhibit distinct pH tolerance ranges and optimal pH values. As shown in Figure 7, under conditions of a culture temperature of 30 °C, an inoculum size of 10%, and an initial diflufenican concentration of 40 mg/L, strain 88-1 failed to grow in the diflufenican-containing inorganic salt medium at pH 5.0, and the corresponding degradation rate remained at only trace levels relative to natural attenuation. In contrast, within the pH range of 6.0 to 8.0, both cell density and degradation efficiency increased significantly with rising pH, from 0.83 × 10^6^ CFU/mL and 18.64% to 2.5 × 10^6^ CFU/mL and 43.94%, respectively. When the medium pH was increased to 9.0, both the growth and degradation performance of strain 88-1 showed no significant difference compared to the peak values observed at pH 8.0.

#### 3.3.5. Effects of Inoculation Amount and Growth of Strain 88-1 on Degradation

**Figure 8:**
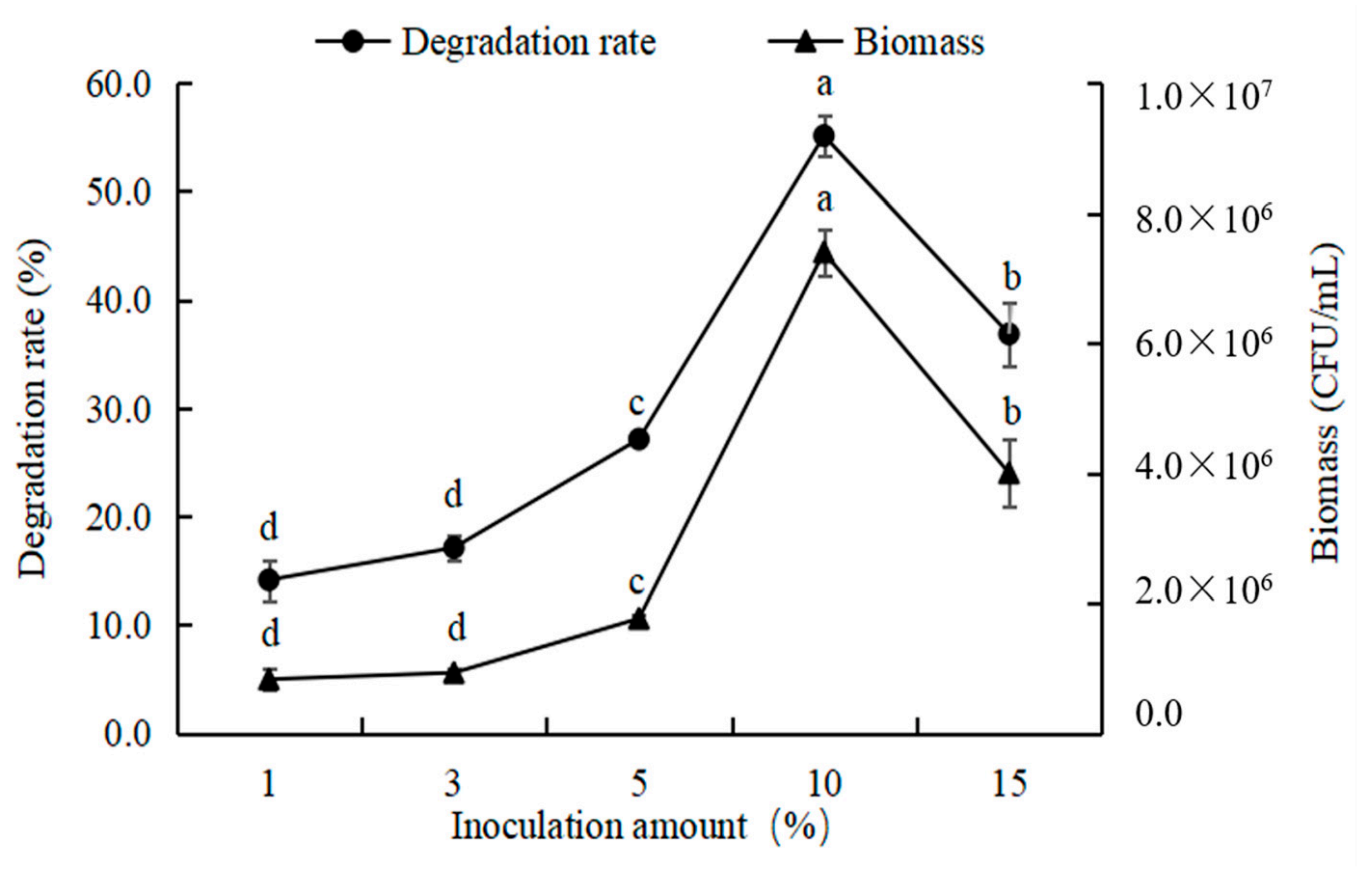
Effects of inoculation amount on the degradation rate of diflufenican and growth of strain 88-1 (Different lowercase letters indicated significant differences (α = 0.05) analyzed by Duncan’s multiple range test).

**Figure 9:**
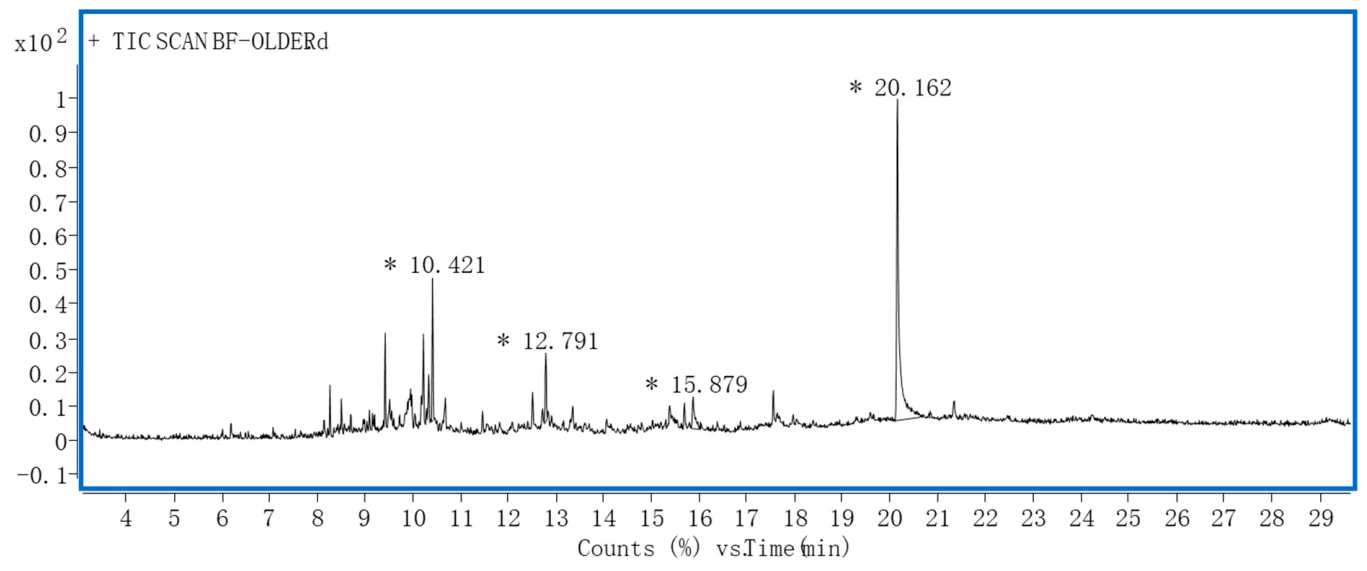
Total ion chromatogram (TIC) of diflufenican degradation products.

The inoculation amount of a microbial strain is generally closely correlated with its substrate degradation efficiency and growth kinetics. In this study, the effects of five inoculation levels (1%, 3%, 5%, 10%, and 15%) on the growth of strain 88-1 and its capacity to degrade diflufenican were evaluated under controlled conditions: a culture temperature of 30 °C, initial medium pH of 8.0, and an initial diflufenican concentration of 40 mg/L. Results are presented in Figure 8. As shown in Figure 9, both the bacterial growth and diflufenican degradation rate increased with inoculation amount within the range of 1% to 10%. At 1% inoculation, diflufenican degradation reached only 14.14%, with a cell density of 0.83 × 10^6^ CFU/mL. In contrast, at 10% inoculation, degradation efficiency peaked at 55.11%, and cell density reached a maximum of 7.4 × 10^6^ CFU/mL, with statistically significant differences compared to other treatment groups. However, when the inoculation level was further increased to 15%, both cell growth and degradation performance declined significantly relative to the 10% inoculation group.

#### 3.3.6. Degradation Pathway of Diflufenican by Strain 88-1

**Figure 10:**
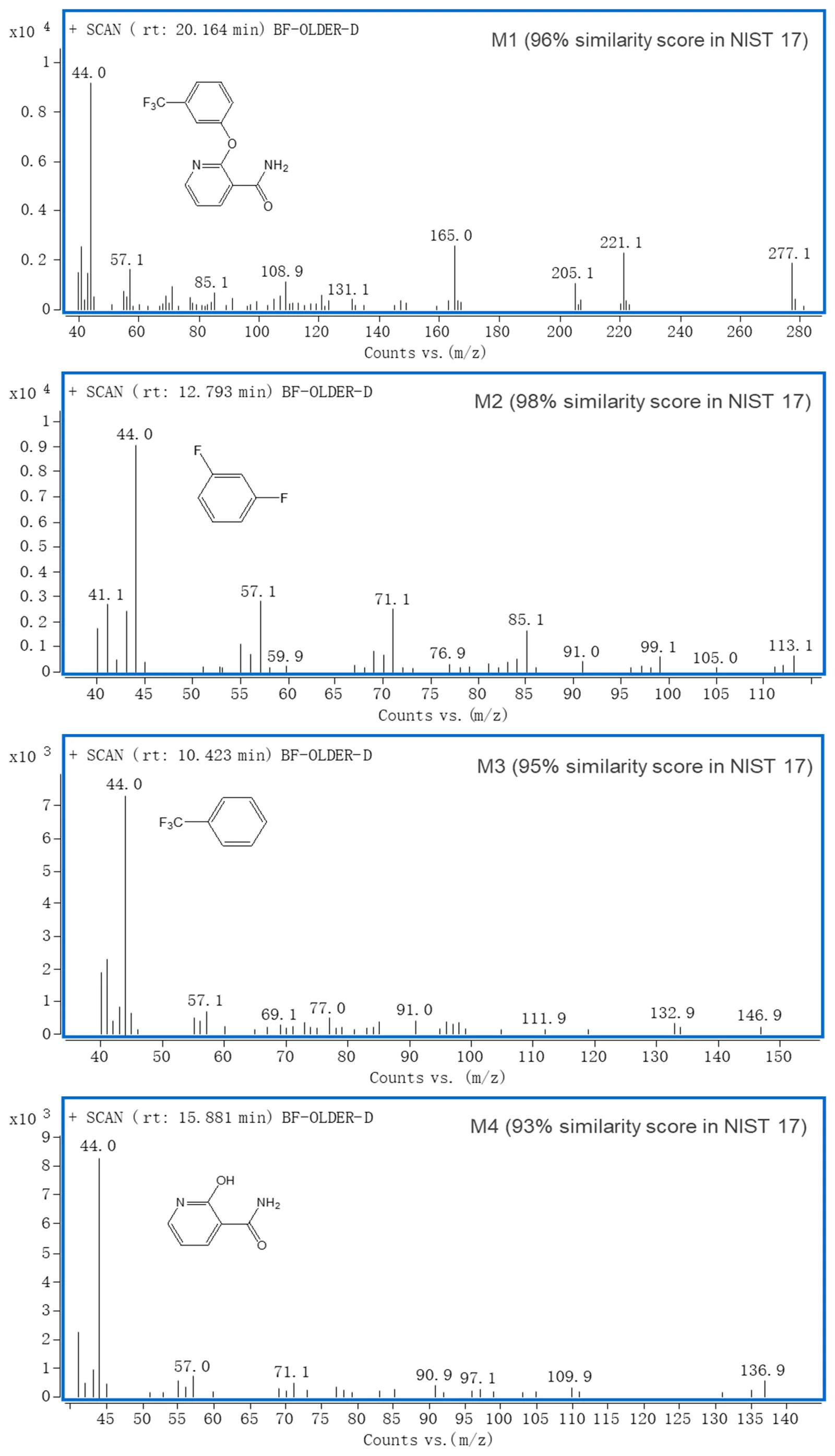
Mass spectrum of degradation metabolites of diflufenican(M1: 2-(3-(trifluoromethyl)phenoxy)pyridine-3-carboxamide; M2: 1,3-difluorobenzene; M3: 1-(trifluoromethyl)benzene; M4: 2-hydroxypyridine-3-carboxamide).

**Figure 11:**
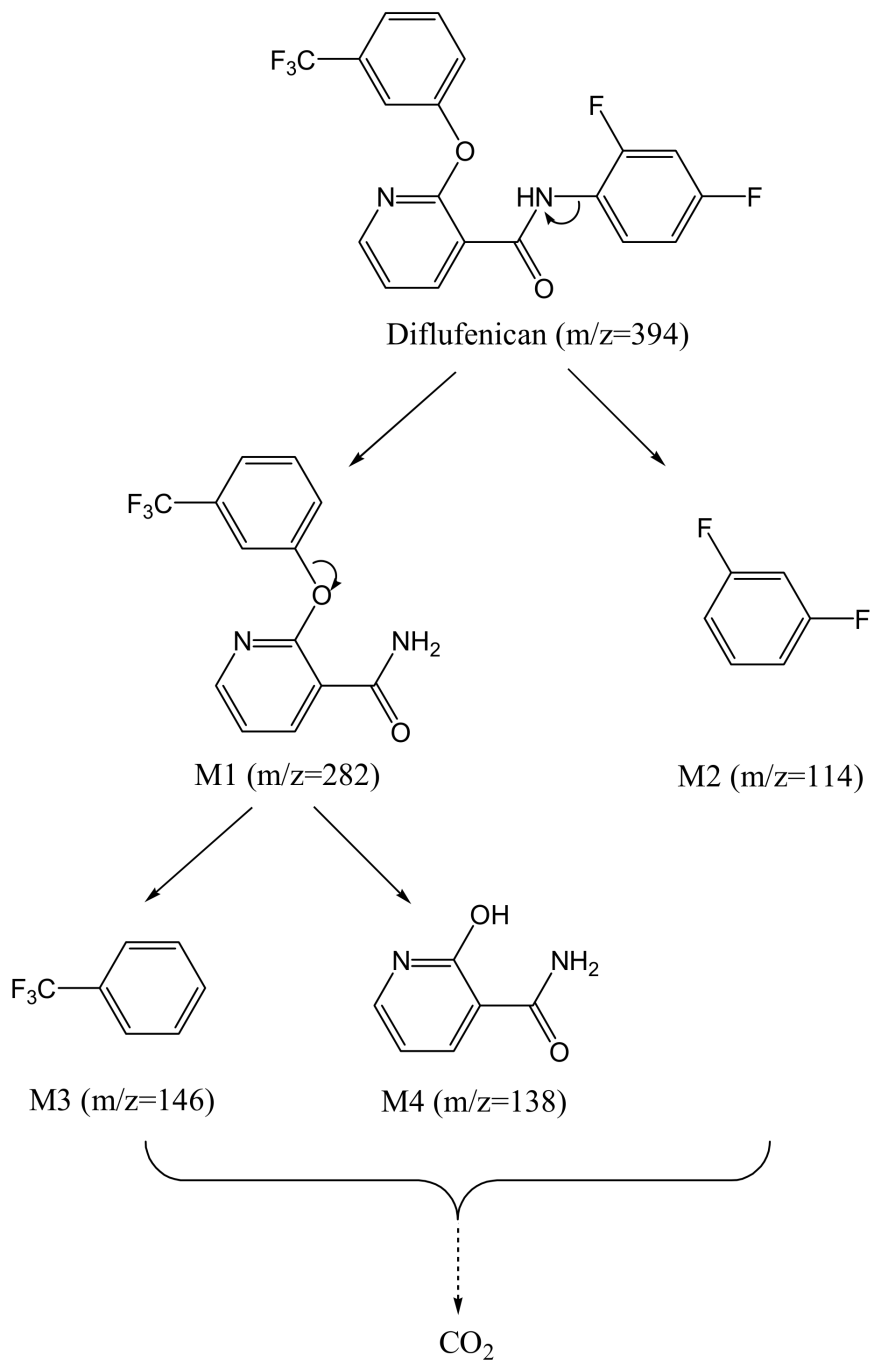
The degradation pathway of diflufenican by strain 88-1.

Selected degrading strains are generally capable of specifically accelerating the degradation of target substrates in the environment. In this study, the degradation and metabolic behavior of diflufenican in an inoculated inorganic salt medium was examined under controlled conditions: a culture temperature of 30 °C, medium pH of 8.0, an initial diflufenican concentration of 40 mg/L, and an inoculation ratio of 10%. At least four degradation metabolites derived from diflufenican were unequivocally identified in the total ion chromatogram (Figure 9) of the acetonitrile extract from the strain 88-1-mediated inorganic salt medium. The detailed mass spectrometry analysis results of these four degradation metabolites are presented in Figure 10. The proposed degradation pathway of diflufenican by strain 88-1, derived from these data, is illustrated in Figure 11. As shown in Figure 11, diflufenican initially undergoes amide bond cleavage under the action of strain 88-1, yielding metabolite M1 (m/z 281; identified as 2-(3-(trifluoromethyl)phenoxy)pyridine-3-carboxamide and metabolite M2 (m/z 113; identified as 1,3-difluorobenzene). Subsequently, M1 undergoes further ether bond cleavage to produce metabolite M3 (m/z 146; identified as 1-(trifluoromethyl)benzene) and metabolite M4 (m/z 136; identified as 2-hydroxypyridine-3-carboxamide). It is inferred that all intermediate metabolites are ultimately mineralized completely to CO_2_.

## 4. Conclusions

In this study, a bacterial strain designated as 88-1, capable of degrading diflufenican, was isolated from soil samples collected from a wheat field with a long-term history of diflufenican application. The strain was identified as *Enterobacter hormaechei* based on morphological, physiological, and biochemical characteristics, as well as gene sequence analysis.

This study demonstrates that the degradation rate of diflufenican by *Enterobacter hormaechei* strain 88-1 increases progressively with prolonged incubation time within a defined period, a trend consistent with findings reported by Ksiazek-Trela et al. [14]. The relationship between the initial diflufenican concentration and its degradation behavior in this study aligns well with the results of Liu et al. [16] and Wang et al. [17], all indicating that the degradation efficiency rises with increasing substrate concentration within an optimal range. However, at excessively high concentrations, diflufenican exerts inhibitory effects on the growth of strain 88-1, consequently reducing its degradation capacity. The optimal degradation temperature was determined to be 30 °C, which is in agreement with most published studies on bacterial herbicide degradation, suggesting that this temperature represents a favorable condition for the growth and metabolic activity of diverse degrading microorganisms [11,12,16,17,18,19,20]. Strain 88-1 exhibited robust growth and metabolic activity across a pH range of 6.0–9.0, but failed to grow at pH 5.0, indicating a preference for neutral to slightly alkaline conditions. This pH adaptability is consistent with previous reports on *Enterobacter hormaechei* strains used in surfactant production [21] and carotenoid degradation [22], where an initial pH of 6.0 was also found to be suitable. Nevertheless, compared to other members of the *Enterobacter* family that exhibit broad pH tolerance (pH 3.0–11.0) [18,23], strain 88-1 displays a relatively narrower pH adaptation range. Its optimal pH of 8.0 is comparable to values reported for microbial degradation of other herbicides [13,16,17,18,19], highlighting a common physiological preference among certain degrading bacteria. The influence of inoculum size on degradation efficiency followed a unimodal pattern—increasing initially and then declining at higher doses—a phenomenon similarly observed by Hou [20] and Neetha et al. [24] This trend may be attributed to insufficient cell density and slow proliferation at low inoculation levels, whereas excessive inoculum leads to rapid population expansion during cultivation, intensifying competition for essential resources such as nutrients and dissolved oxygen, thereby impairing microbial growth and metabolic performance. Furthermore, strain 88-1 rapidly transformed diflufenican into multiple metabolites—including 2-(3-trifluoromethylphenoxy) nicotinamide and 2,4-difluorobenzene—within 24 h. These findings are qualitatively consistent with the soil metabolite profiles of diflufenican reported by Gu et al. [3]; however, the present study identified a broader spectrum of intermediate metabolites, suggesting that strain 88-1 possesses a more comprehensive catabolic pathway.

## 5. Discussion

The long-term and extensive use of the persistent herbicide inevitably poses potential risks to soil environments, ecosystems, and food safety. Microbial degradation, which offers advantages such as non-toxicity, absence of residual byproducts, and effective prevention of secondary pollution, has become a promising strategy for removing pesticide residues from agricultural soils and supporting sustainable agricultural development [25]. For amide herbicides, numerous degrading strains have been isolated from diverse ecological habitats, including farmland soils, wastewater discharge outlets of pesticide manufacturing facilities, and activated sludge, primarily belonging to bacterial genera such as *Pseudomonas* sp. [26,27], *Bacillus* sp. [28,29], *Sinorhizobium* sp. [30,31], and *Enterobacter* sp. [18,32]. However, these reported strains are predominantly involved in the degradation of chloroacetamide herbicides—such as acetochlor, butachlor, and metolachlor—whereas degrading microorganisms capable of metabolizing pyridine-substituted anilide herbicides like diflufenican remain rarely documented. Growth characteristics vary among different degrading bacteria; during the degradation of herbicide residues, key factors—including incubation time, initial herbicide concentration, temperature, pH, and initial inoculum size—primarily influence degradation efficiency by affecting microbial biomass accumulation.

*Enterobacter hormaechei* 88-1 exhibits significant potential as an efficient diflufenican-degrading bacterium and could serve as a promising microbial agent for bioremediation of pesticide-contaminated soils, offering favorable prospects for environmental application. Nonetheless, its degradation efficacy, ecological safety, and key engineering parameters under real-world agricultural conditions require further systematic investigation.

## Figures and Tables

**Table 1:** Physiological and biological characterizations of strain 88-1.

Number	Detection Index	Strain 88-1
1	Gram Staining	−
2	Starch Hydrolysis test	−
3	Gelatin Hydrolysis test	+
4	Methyl red test	−
5	Voges-Prokauer test	+
6	Hydrogen Sulfide test	−
7	Urea test	−
8	Indol test	−
9	Citrate test	+
10	Deoxyribonuclease test	−

Note: + indicates a positive reaction; − indicates a negative reaction.

**Table 2:** Comparison results of the gene sequence of strain 88-1 against the NCBI database.

Description	Max Score	Total Score	Query Cover	E Value	Match Degree (%)	Accession
*Enterobacter hormaechei* strain PG20180049 chromosome, complete genome	2702	1463	100	0.0	99.66	CP0432 26.1
*Enterobacter hormaechei* strain 2024CK-00067 chromosome, complete genome	2708	1466	100	0.0	99.73	CP1500 11.1
*Enterobacter hormaechei* strain C15 chromosome, complete genome	2713	1469	100	0.0	99.80	CP0424 88.1

## Data Availability

The original contributions presented in this study are included in the article. Further inquiries can be directed to the corresponding authors.
